# Acceptability of insecticide treated nets in an urban setting in a malarious area: Application of theoretical framework of acceptability

**DOI:** 10.1371/journal.pgph.0006120

**Published:** 2026-08-21

**Authors:** Sosina W. Tilahun, Berhan Tassaw, Guofa Zhou, Dagim Tefera, Ahmed Ali, Daibin Zhong, Ming-Chieh Lee, Randy E. David, Wakgari Deressa, Guiyun Yan, Daniel M. Parker, Werissaw Haileselassie

**Affiliations:** 1 School of Nursing and Midwifery, Addis Ababa University, Addis Ababa, Ethiopia; 2 School of Public Health, Addis Ababa University, Addis Ababa, Ethiopia; 3 Program in Public Health, University of California, Irvine, California, United States of America; 4 Department of Public Health, Arbaminch College of Health Sciences, Arbaminch, Ethiopia; 5 School of Medicine, Wayne State University, Detroit, Michigan, United States of America; Tulane University School of Public Health and Tropical Medicine, UNITED STATES OF AMERICA

## Abstract

Malaria has long been considered a rural disease. However, rapid urbanization, the expansion of urban malaria vectors, and the subsequent, ongoing surge in malaria cases across sub-Saharan Africa, including Ethiopia, underscore the need to address malaria in urban contexts. This study therefore aims to explore the acceptability of insecticide-treated bed nets (ITNs) in an urban setting using the theoretical framework of acceptability. Eight Focus Group Discussions (FGDs), comprising a total of 48 participants, were conducted in the four Kebeles of Batu town, East Shoa, Oromia, Ethiopia. A semi-structured interview guide, developed based on the constructs of the Theoretical Framework of Acceptability (TFA), was used to facilitate the discussions. Data were analyzed using content analysis with a deductive approach, focusing on the seven components of the TFA. All data were managed with Atlas. ti software, version 9. Participants demonstrated a strong understanding of ITNs, expressing positive attitudes and noting a reduction in malaria cases following their introduction. However, concerns regarding the ethicality of ITNs emerged, particularly in relation to sexual assault and the discomfort experienced by pregnant women due to chemical exposure, which was perceived as violating the principle of non-maleficence. Participants also voiced dissatisfaction with the short height of currently available ITNs and expressed mixed feelings about their shapes; women preferred circular shaped nets, while men favored squared ones. In addition, they reported burdens such as adverse skin and respiratory reactions. Female participants, in particular, emphasized the increased household chores associated with handling the ITNs as a part of their morning routine. In conclusion, ITNs can be considered an appropriate intervention in controlling urban malaria. However, practical challenges must be addressed to improve their acceptance and usability.

## Author summary

This study is the first of its kind to introduce a structured framework for assessing the acceptability of malaria interventions, facilitating the transition from prototypes to widespread use and ensuring community ownership and utilization. It provides a systematic and scientifically sound approach to investigating intervention acceptability for malaria, addressing a gap in the existing literature. Furthermore, it provides a rare urban-level assessment of ITN acceptability, generating evidence from an urban perspective. The increasing malaria transmission in cities amplifies the relevance of this research. In addition, the findings have practical implications for strengthening malaria management program.

## Introduction

For a long time, malaria was viewed primarily as a disease of rural areas, attributed to the breeding of *Anopheles mosquitoes* that transmit *Plasmodium* species commonly found in such environments [1]. This reality stems from the inability of rural populations to afford protective measures such as improved housing and a clean environment, which could mitigate the risk of contracting the disease. Inadequate diagnosis and treatment further exacerbate their susceptibility to malaria [2]. Contrary to this notion, research has revealed that urban areas also play a significant role in the global incidence of malaria, contributing between 6% and 28% to the estimated annual malaria burden worldwide [3,4].

Multiple factors have been identified as contributing to malaria transmission within urban environments. These include pollution attributed to poor waste management in urban areas, which increases risks for malaria transmission [5]. Poor housing, poor drainage, deep inequalities in vector control between urban and rural areas increase the potential risks for large outbreaks [6]. Additionally, a study has indicated that irrigated rice paddies, vegetable gardens, trenches, and irrigation wells serve as productive breeding sites for mosquitoes [7]. Thus, urban agricultural practice may create a man-made breeding site for the mosquitoes. Furthermore, unplanned and rapid urban growth exacerbates malaria risk by contributing to overcrowded, substandard housing and peri-urban sprawl, which facilitate vector breeding. Human density, housing, window screens and light condition in the urban may affect resting and biting behavior of malaria vector species with marked implication for malaria transmission [5,7,8].

The phenomenon of rapid urbanization in several African nations is primarily driven by individuals migrating from rural areas in search of improved employment and educational opportunities within urban centers [9–11]. Ecological changes associated with urbanization in peri-urban and urban slums, can increase urban malaria transmission by introducing infected individuals, creating new mosquito breeding habitats, and enabling malaria vectors to adapt to urban environments [12]. Increased movement of people nationally and internationally through migration, displacement or employment increases the rate of new malaria parasite strains being spread. Recognizing these dynamics, Word Health Organization (WHO) called for a concerted global response to urban malaria, emphasizing the differences in malaria transmission patterns and disease burden between rural and urban settings and advocating for targeted approaches in urban ecosystems [13].

In Ethiopia, although malaria is primarily a rural disease, a significant proportion of urban residents are affected, and human activities in urban areas play an important role in creating mosquito breeding sites near to human dwellings [2]. As in many developing countries, Ethiopian towns face challenges such as inadequate housing, poor sanitation facilities, insufficient drainage systems, limited access to quality healthcare services, and marked economic disparities. These factors, either individually or in combination, create favorable conditions for malaria transmission within urban areas [14]. In Ethiopia, urban malaria was estimated to account for approximately 3.9-5.3% of the country’s total malaria burden between 2006 and 2011 [2,15]. By 2019, this proportion had risen to 15.3% [14]. This trend indicates that malaria transmission is not confined to rural settings, which points to the need for comprehensive strategies to combat the disease in both urban and rural contexts.

The use of Insecticide Treated Nets (ITNs) is crucial to the prevention, control, and elimination of malaria [16]. Despite efforts to promote ITN ownership and utilization of ITNs, a persistent mismatch remains between ownership and actual use, particularly in Sub-Saharan Africa (SSA) [17,18]. The best practice for developing innovative health interventions calls for the assessment of local acceptability of the intervention. Implementing cost-effective and appropriate interventions requires a comprehensive understanding of how these interventions are perceived and adopted by the urban population. Moreover, assessing acceptability is vital, as it determines the extent to which proposed tools and interventions are embraced by the target communities [19]. Failure to evaluate acceptability can impede the ownership and utilization of interventions, thereby hindering the transition from prototype to widespread use.

In Ethiopia, exploration of ITN acceptability in urban areas has been limited due to lack of research approaches and standardized tools, as urban malaria has historically been underestimated and overlooked. Therefore, the aim of this study was to assess ITN acceptability in a malaria-endemic urban setting of Batu/Ziway, Ethiopia, to generate evidence for the design of tailored interventions to ensure ITN acceptability among urban communities.

### Theoretical framework

ITN acceptability was explored using the Theoretical Framework of Acceptability (TFA), which provides a structured, multifaceted model. The model consists of seven constructs: affective attitude (how individuals feel about the intervention), burden (the perceived amount of effort required from an individual in order to participate in the intervention), ethicality (the extent to which the intervention fits into an individual’s value system), intervention coherence (whether the individual understands the intervention or how it works), opportunity cost (what has to be given up by the individual in order to participate in the intervention), perceived effectiveness (the extent to which the intervention is perceived to achieve its purpose), and self-efficacy (how confident the individual is to participate in the intervention) [20].

The TFA offers a comprehensive structure for assessing multiple dimensions of intervention acceptability, enabling policymakers to identify which domains require further attention and why [20].

## Methods

### Study setting

Batu town, formerly known as Zeway, is situated in the central region of Ethiopia, approximately 165 km south of Addis Ababa, the capital city of Ethiopia. It is located within the Ethiopian Rift Valley. Batu town is positioned at 7° 56’ 03"N latitude and 38° 42’ 56” E longitude. Its average altitude is 1657 m above sea level. The town encompasses four kebeles, namely: Betele, Melka Shalo, Melka Wafoki, and Dembel, which are local government administrative units below the district level. The total population of Batu is 78,784, consisting of 40,180 men and 38,604 women. Ziway has a fairly warm climate with a mean annual temperature of 21°C, while the area experiences an annual rainfall ranging between 700 and 800 mm. The humidity is around 53%. The main rain season extends from June to September, and the short rain season begins in March and extends to May. Malaria transmission in the Batu area is generally characterized as unstable, occurring seasonally. The peak transmission period takes place between September and November, while another transmission period occurs between April and May during the short rainy season.

### Study design and period

A qualitative descriptive design was conducted to explore the acceptability of ITNs in the malaria-endemic urban setting of Batu town, Oromia, Ethiopia, from November 01 to November 30, 2024. Focus groups were identified, and moderated discussions were conducted to gather participants’ opinions, experiences, and insights regarding ITN acceptability.

### Recruitment

Separate focused groups of women and men, each consisting of six participants, were recruited from the four kebeles of the study area. Inclusion criteria required participants to be an adults who had lived in the area for at least the last five years, had used ITNs consistently for at least the last two years to prevent urban malaria, and were proficient in Amharic and Afan Oromo. Finally, those who were able and willing to provide full informed consent were included in the study. A cover letter containing information about the study, along with an ethical approval letter from the Institutional Review Board of the College of Health Sceicnes at Addis Ababa University, was submitted to the town administration bureau one week prior to data collection. Following administrative approval, letters were sent to the community leaders of the four kebeles, and separate focused groups of men and women with diverse backgrounds were formed from each kebele.

Participant recruitment was conducted outside health centers, hospitals, or social gatherings to ensure inclusion of community members who might otherwise been missed. In each Kebele, community leaders assisted in identifying households with eligible adults. From these households, participants were purposively selected to capture diversity in age, educational background, religion, employment, and marital status [21]. Each individual was approached directly at home, typically in the early morning before leaving for work, informed about the study’s objective, and invited to participate. Consent was obtained at this stage, and there was no recorded participants’ refusal or dropout. This process resulted in two independent focus groups per kebele: six women and six men, ensuring gender balance and representation.

Socio-demographic characteristics of the participants were collected prior to the discussions using an interviewer-administered questionnaire. Each FGD was audio-recorded, transcribed, and translated verbatim into English.

### Data collection

The interview guide was originally developed by SWT and WH, based on the seven domains of the Theoretical Framework of Acceptability (TFA) [20]. To ensure cultural appropriateness, the guide was translated to Amharic and Afan Oromo, the two widely spoken languages in the town. Translation was performed by an independent expert who is fluent in Amharic, Afan Oromo, and English and familiar with qualitative research. A back translation was subsequently conducted by an independent translator who is an expert in the languages to check consistency and accuracy. Discrepancies were resolved after thorough discussion among the translators and the research team to preserve the intended meaning of each construct. The final Afan Oromo and Amharic versions were used during the FGDs, accordingly (S1 Text).

Prior to data collection (interview), the interview guide was tested among a small group of participants who share similar context with the actual study participants. The pilot test assessed the clarity and phrasing of the questions, cultural sensitivity, natural flow, language and translation, and timing of the FGD guide. Furthermore, it enabled the research team to evaluate note-taking and consent procedures. The feedback from the pilot test was used to refine natural flow, probing, and clarity.

A total of eight FGDs, two from each kebele, were conducted with 48 in quiet outdoor areas near participants’ homes, chosen to avoid noise and sun discomfort. The duration of each FGD ranged from 1 to 2 hours and 46 minutes, with an average of 1.5 hours. Once internal saturation was reached for a domain, the point at which a specific TFA domain after successive FGDs produced no new insights and participant responses became repetitive [22], the facilitator proceeds to the next construct. Overall saturation regarding the *ethicality domain* was declared during the final FGD.

### Researcher positionality and reflexivity

The focus group discussions were conducted by the research team members with prior experience in qualitative methods. SWT, a public health researcher with experience in qualitative study, facilitated discussions, while DT, a public health researcher, served as a note-taker. Multiple efforts were undertaken to minimize bias throughout the study. Reflexive notes were maintained in the research process to ensure the researchers’ transparency. Triangulation of data from gender segregated groups and Kebeles was carried out, while peer debriefing was conducted with other research teams who have experience in qualitative research and were not involved in the data collection. They provided critique and support in coding decisions. The facilitator (SWT) emphasized neutrality during discussion, encouraged diverse viewpoints and probed for alternative perspectives to reduce interviewer influence.

Quotations were carefully edited for readability without altering meaning, further minimizing interpretive bias. Reporting adhered to the Consolidated Criteria for Reporting Qualitative Research (COREQ) checklist [23], ensuring transparency in the study design, recruitment, data collection, and analysis (S1 File).

### Data analysis

Theory-informed content analysis was applied to analyze the qualitative data collected during the FGDs. The analysis was guided by the constructs of TFA to extract meaning and organize the findings against the domains [20,24]. All audio recordings were transcribed in the languages used during the discussions (Afan Oromo or Amharic). The transcripts were then translated into English, a process that safeguarded meaning equivalence across languages and minimized interpretive bias. Member checking was done through sharing transcripts with the participants of the study. Accuracy in interpretation was ensured through techniques that can preserve meaning like using skilled bilingual translators, conducting back-translation, review of translations by the research team members.

The data were coded independently by two coders (SWT and BT) using the domains of the TFA as a guiding framework. Inter-coder verification was performed to ensure that the assigned codes accurately reflected the content of participants’ responses. Codes were then organized under the relevant TFA domains, and any variations between coders were discussed until consensus was reached on the most appropriate domain assignment by discussing with other research teams. This iterative process enhanced reliability and minimized subjective bias in interpretation. The coded themes and sub-themes were subsequently exported from ATLAS.ti version 9 into Microsoft Word Office for further review and interpretation (S1 Table).

### Ethical considerations

Ethical clearance was obtained from the Institutional Review Board (IRB) of the College of Health Sciences at Addis Ababa University under protocol number 010/23/SPH. Permission was obtained from the town administration. Then written informed consent was obtained from each study participant and additionally audio recorded. Participant confidentiality was maintained by removing all possible identifiers, including names of the participants. All methods adhered to relevant guidelines and regulations.

## Results

### Socio-demographic characteristics of study participants

A total of eight FGDs involving 48 individuals were conducted across four Kebeles in Batu town. (Table 1). Half of the participants were female, and approximately 46% were older than 35 years. The study adequately represented individuals with diverse educational backgrounds, religions, marital statuses, and occupations. This diversity provided a strong empirical foundation for the opinions, experiences, perspectives, and insights that emerged.

**Table 1:** Socio-demographic characteristics of study participants in Batu town, East Shoa, Oromia, Ethiopia (n = 48).

Variables	Betele (n = 12)	Melka Shalo (n = 12)	MelkaWafoki (n = 12)	Dembel (n = 12)
**Mean Age**	32.1	33.8	46.1	30.3
≤ 35 Years old	8	9	2	7
> 35 Years old	4	3	10	5
**Gender**				
Female	6	6	6	6
Male	6	6	6	6
**Religion**				
Orthodox	4	4	6	3
Muslim	3	4	3	3
Protestant	3	3	2	4
Catholic	2	0	1	1
Others*	0	1	0	1
**Marital Status**				
Single	5	3	3	5
Married	3	6	4	4
Separated (No legally divorced)	1	1	2	2
Divorced	1	2	2	1
Widow	2	0	1	0
**Level of Education**				
No formal Education	0	2	3	2
Primary school (1–8)	1	7	3	1
High school (9–12)	1	1	1	1
Occupational Training	6	0	3	2
Diploma/Degree or above	4	2	2	6
**Occupation**				
Employed	11	8	7	6
Unemployed	0	3	4	4
Retired	1	1	1	2

*Adventist, Waqefatta.

### Theoretical framework of acceptability

#### Positive and Negative feelings towards an ITN.

Participants expressed a variety of feelings regarding insecticide-treated nets (ITNs). They described positive feelings on physical protection, emotional comfort, and trust.

*When I think of the ITN, it brings me comfort, knowing that I don’t have to worry about contracting malaria. It allows me to sleep peacefully, reassured that I’m protected while resting beneath it.* (**R6, Female, Betele Kebele**)

Furthermore, participants reported experiencing disturbed sleep patterns when they didn’t use ITNs. They emphasized the contribution of ITN to good sleeping quality, stating:

*When you use an ITN, you can enjoy a peaceful night’s sleep. Without ITN, mosquitoes will definitely keep you awake the whole night.* (**R4, Female, Melka Wafoki Kebele**)

Participants reported the negative feelings related to the height of the ITN. They experienced a sense of unease, which was linked to their reluctance to use ITNs altogether.

*Nowadays, the size of the ITN is smaller and shorter in height. When it is like that, it makes you anxious when you sleep under it. I am not going to lie to you; I don’t use it, as it makes me feel like I am losing breath.* (**R1, Male, Dembel Kebele)**

Participants expressed mixed feelings regarding the shapes of insecticide-treated nets (ITNs). Although ITNs are available in various forms, the circular and rectangular (four-sided) designs are the most commonly found on the market. Female participants reported feeling more comfortable with the circular-shaped ITN, appreciating its simplicity in handling and its suitability for different living conditions, such as rental homes.

*I like the circular one because, when living in a rental house, you can’t use nails to hang things on the walls. Since the circular design doesn’t require nails, it’s much more comfortable to use. Landlords don’t want the house damaged with nails.* (**R1, Female, Dembel Kebele**)

*So, the circular one also keeps me from getting bored in the process of using an ITN.* (**R1, Female, Betele Kebele)**

Furthermore, the aesthetic appeal of the four-sided ITN was also emphasized,

*And if you can make the ITN in the shape of the bed, like a honeymoon house, it would also have beauty that way*. **(R2, Female, Dembel Kebele)**

However, the need for multiple points of attachment to the wall made the four-sided ITN less acceptable for use in rental homes, as it involves more effort to hang over the bed.

*Nowadays, some landlords don’t want you to put nails into the walls. The four-sided ITN needs to be hung at four points in the corners of the house. That is a bit challenging, as it requires at least four people to hang it.* (**R5, Female, Betele Kebele**)

Furthermore, participants shared a range of concerns regarding ITN use, which they attributed to the chemical on the nets. They complained about the odor, as well as its itching and burning sensations caused by the insecticide treatment.

*When I sleep under it, the smell of the ITN really bothers me. I just can’t stand that odor.* (**R6, Female, Melka Shalo Kebele**)

*The reason people do not like ITN is that if the chemical touches you, it burns. It burns severely, sometimes for two or three weeks, and may even turn into a wound.* (**R3, Female, Melka Wafoki Kebele**)

Additionally, the odor of the chemicals was reported to cause allergic reactions, leading to headaches among some participants.

*Sometimes, I wash the ITN to remove the chemical so that I don’t smell it. I use it after washing it away. I cannot use it with the chemical on, it is difficult for me.* (**R4, Female, Melka Wafoki Kebele**)

*Even because of the smell, one can develop a headache. I have seen that happen. So, the smell causes headaches.* (**R3, Female, Melka Shalo Kebele**)

The participants also emphasized that children, often unaware of the chemical treatments applied to the nets, may not take the necessary precautions while sleeping, thereby increasing their risk of adverse effects.

*Even the chemical on it (an ITN) can kill a newborn child.* (**R6, Male, Dembel Kebele**)

While most participants recognized that ITNs provide protection against malaria and various disease-causing flies, some expressed reservations about sleeping under them. These reservations could reduce interest in ITNs, ultimately hindering their acceptance within the community. To address this issue, participants suggested providing ITNs in different shapes and raising awareness about their use at various levels of the community.

*We also use the four-sided, but when it becomes easy and comfortable, you will use it more happily. Because if it becomes comfortable, I don’t think there would be anyone hating ITNs.* (**R6, Female, Melka Shalo Kebele**)

*And also, people hates using ITNs because they don’t have training on how to use them.* **(R5, Male, Betele Kebele)**

#### The perceived amount of effort that is required to use an ITN.

This construct explored the specific circumstances in which ITN use was considered burdensome. Participants particularly highlighted the difficulty of sleeping under ITNs during the daytime and the summer season.

*Now you cannot sleep under an ITN during the daytime; I would rather prefer to be bitten by mosquitoes. It is better during the night. Since the weather is cooler at night, you are better off sleeping then.* (**R6, Female, Betale Kebele**)

They expressed that the already uncomfortable weather conditions were exacerbated by the net, leading to a feeling of being trapped in an oppressive environment. These dual challenges not only affect quality of sleep but also contribute to a general sense of unease and discomfort.

*It difficult to use during summer because it is hot. With the heat, adding the ITN makes you unable to breathe.* **(R6, Female, Dembel Kebele)**

Furthermore, participants elaborated on the particular burdens that sleeping under ITNs imposes on pregnant women. The combination of physical discomfort, physiological strain, and emotional stress creates a challenging sleeping environment for pregnant women while using ITNs.

*Now, if you see a pregnant woman, she breathes for herself and for her child, right? As I told you earlier, since ITN makes breathing difficult and stressful, I think it is hard for her to use it.* (**R2, Female, Melka Shalo Kebele**)

Participants emphasized the importance of providing clear instructions and orientation before using ITNs to facilitate the safe incorporation of them into daily routines. They also suggested practical modifications to enhance usability, such as increasing the size of the nets to prevent direct contact with the skin and thereby reduce potential skin reactions.

*The instructions on how to use an ITN are already included in the manual, both in English and Amharic. You can read there before hanging. Those who cannot read may face challenges. If you directly use it after unpacking, it is very difficult. At least it needs to be kept in open air for two days, the manual says. When I first used ITN, the chemical on it burned me for three consecutive days. No one will use it after the first experience if you don’t read the manual.* (**R3, Female, Dembel Kebele**)

#### The extent to which the ITN has a good fit with an individual’s value system.

Participants strongly supported the intervention and emphasized that using ITNs aligns with their cultural values and practices.

*ITN is life.* (**R2, Female, Betele Kebele**)

However, gender and marital concerns within the community were identified as reasons some participants were hesitant to use ITNs. Cultural norms dictate that men and women cannot share a bed unless they are married. This concern was particularly emphasized in connection with ITN distribution practices. In Ethiopia, ITN deployment often means that a household with eight family members receives only two nets, which may result in individuals of opposite sexes sharing the same ITN.

*If they give you one ITN for a family of four, you make the family of four sleep together. But who are those family members you are making sleep together? These days, the time is not good; making a boy and a girl sleep together is a bad decision.* (**R1, Female, Melka Shalo Kebele**)

The majority of participants also expressed concerns about the compliance of pregnant women with ITN use, viewing it as potentially unethical to compel a pregnant woman to sleep under ITNs. They highlighted that inhaling the chemicals used in ITNs may pose risks for pregnant women, who are responsible for their own health and that of their unborn child.

*I don’t think it is fair to compel pregnant women to sleep under ITNs. Because they contain chemicals, she may inhale them. And also when she wants to exhale, it also becomes a problem for her. So, personally, I don’t support this.* (**R6, Male, Dembel Kebele**)

#### The extent to which participant understands the ITN and how it protects them from malaria.

Generally, participants demonstrated a good understanding of what ITNs are and how they function, including their protective role against malaria. They showed awareness of ITN’s dual protective features, which include both chemical and physical protection. Participants acknowledged that the net prevents mosquito bites by creating a physical barrier when people sleep under it.

*If the ITN does not get into chemicals, it may prevent mosquitoes, but the mosquito will not disappear. But if the ITN has the chemical, it would kill the mosquitoes.* (**R2, Male, Betale Kebele**)

Furthermore, participants clearly understood the multipurpose benefits of ITNs. In addition to protecting against malaria-carrying mosquitoes, they recognized that ITNs also shield from other disease-carrying flies and even household rats, which are often found in rural and less hygienic environments. For example, participants noted that ITNs can deter flies that cause discomfort and disrupt sleep, thereby contributing to a more restful and healthier sleeping experience.

*ITN prevents not only mosquitoes but also other insects, right? Now when we sleep under ITN, various insects and worms cannot get into our mouth or bite us. So, it helps us a lot.* (**R4, Female, Dembel Kebele**)

Participants also demonstrated a clear understanding that the long-lasting effectiveness of ITNs is largely due to the chemical insecticides embedded in the nets.

For DDT (***Dichloro-Diphenyl-Trichloroethane***)*, you need to spray it every day. ITNs are preferable due to their lower cost compared to those options. Once you get an ITN, you can use it for a long time, like six months.* (**R1, Female, Melka Shalo Kebele**)

On the other hand, some participants noted that using ITNs may not be easy for everyone. This difficulty influences their understanding of ITNs and, in turn, affects their grasp of the nets’ overall purpose.

*Now if you move in this town, they cover plants with it, they cover cereals with it, and they even collect rubbish with it; they are using it for different unwanted purposes.* (**R5, Male, Melka Wafoki Kebele**)

#### The extent to which benefits, profits, or values must be given up to engage in the ITN intervention.

Participants reported that engaging with ITNs required minimal sacrifices in their daily activities. Specifically, they noted that the actions of hanging the nets before sleep and removing them upon waking were easily integrated into existing household routines.

*At most it may take one or two minutes, not more than that. And tying on the four sides of the bed does not require more than five minutes.* (**R4, Male, Betale Kebele)**

On the other hand, female participants explained how engaging with ITNs on a daily basis interfered with their household responsibilities.

*Now, I have to go to the office in the morning. When you have to prepare breakfast and take care of different things for your children, you need to shuffle these things (****the ITN****).* **(R2, Female, Betale Kebele)**

Additionally, participants explained that the ITN routine at the household level made them less participatory in ITN use.

*Because it makes you stressed with your work routine, and you get bored. So, it adds stress on top of your other routines*. **(R4, Female, Melka Wafoki Kebele)**

#### The extent to which the ITN is perceived to be likely to achieve its aim.

Participants recognized the effectiveness of ITNs among vulnerable populations, including children, pregnant women, new mothers, elderly individuals, and individuals with chronic diseases.

*…In addition, it would be better if a new mother use ITNs. You know why? Most of the time a new mother does not have a chance to use the medication for malaria because she feed breast milk.* (**R2, Female, Betele Kebele**)

Moreover, ITNs were perceived to be particularly effective during the rainy season and at night. Participants highlighted that stagnant water during the rainy season plays a critical role in facilitating malaria transmission by creating breeding grounds for mosquitoes.

*… But, most of the time, it would be better if we used it during winter. Water gets stagnant during winter. There is not much of a problem during summertime, it is during wintertime.* (**R6, Male, Betele**)

Similarly participants indicated that malaria incidence in the community had decreased due to ITN usage.

*Since the ITN was given back, malaria has decreased. Previously it was not like this*. (**R4, Male, Melka Wafoki Kebele**)

*When you get peaceful sleep without the sound of mosquitoes, you come to understand that malaria has decreased.* (**R3, Male, Dembel Kebele**)

On the other hand, participants expressed the belief that the effectiveness of ITNs is largely dependent on the chemical treatment embedded in the net. They emphasized that the primary reason for using ITNs as a preventive measure against malaria is the presence of the chemical, which repels and kills mosquitoes.

*Once the chemical on the ITN wears off, we discard the ITN, as it has no use.* (**R4, Female, Dembel Kebele**)

Additionally, participants shared differing preferences regarding the shape of ITNs, with some favoring the four-sided design over the circular shape and vice versa. These preferences were linked to perceptions of how well each design fulfilled its purpose in the community, such as ease of use, coverage, and overall effectiveness in preventing mosquito bites.

*Once I was given the circular one, and I could not even hang it over the bed. Then I discarded it without properly using it; I didn’t even use it for two days.* (**R2, Female, Betele Kebele**)

#### The participants’ confidence that they can perform the behavior(s) required to participate in the intervention (ITN).

The majority of participants expressed a strong sense of confidence in their ability to utilize ITNs.

*Now in this area, using ITN is a daily routine of the community members. So, it is the simplest activity we do to protect ourselves from malaria. Since it is about life and death, even if it is uncomfortable, we have to do it for our own lives. Because there is nothing better than our life, right?* **(R3, Male, Dembel Kebele)**

Furthermore, participants indicated that self-initiated utilization of ITNs contributed significantly to their confidence levels. They recognized that taking the initiative to use the nets independently not only reinforced their understanding of how to properly implement this preventive measure but also empowered them to feel more in control of their health decisions.

*Rather than the government, it is better when a person becomes aware of it (****the ITN****) and decides to use it. I mean, rather than government. When the government forces you, you just take the net, but you will not use it. So, instead of waiting to hear from the government, it would be better if you initiate yourself and decide to use it.* (**R2, Male, Betale Kebele)**

## Discussion

This study sought to systematically explore individuals’ experiences with insecticide-treated nets (ITNs) as an intervention to combat urban malaria. The objective was to identify the key factors influencing the acceptability of this intervention, thereby enabling informed recommendations for its refinement and supporting its successful implementation. To our knowledge, this paper marks the first application of the Theoretical Framework for Acceptability (TFA) to assess the acceptability of ITNs for malaria prevention in an urban context. By employing the TFA, it provided a structured foundation, rigor, and comprehensiveness that facilitated an in-depth exploration of acceptability to inform programmatic decisions.

This research makes two significant contributions to the existing literature. First, it elucidates the seven constructs of TFA: affective attitude, burden, ethicality, intervention coherence, perceived effectiveness, opportunity cost, and self-efficacy on the acceptability of ITNs. These constructs were derived from a qualitative analysis using focus group discussions among community members of Batu town, who were directly recruited from the residential home. The findings provide insight that enhances our understanding of ITN acceptability in urban settings and has potential to strengthen programmatic decisions aimed at fostering greater acceptance of the intervention. Second, the study outlined specific refinements that could be made to ITNs to further increase their acceptability in urban environments, thereby contributing to the prevention of urban malaria.

Participants were generally positive about using ITNs in urban areas to combat malaria, consistent with broader findings that highlight ITNs as a cornerstone of malaria prevention in such settings [25–27]. However, the size of the ITN influenced comfort, with smaller nets causing more discomfort, while concerns about height arose as participants feared it might trap heat. People prefer ITN depending on its size and durability. A study from Burkina Faso showed a clear preference for nets that were breathable and durable [28]. These findings highlight the role of physical comfortability in shaping attitude toward ITNs. Moreover, a study revealed that environmental discomfort and climate change, like poor ventilation and hot weather, are barriers to proper ITN utilization [29]. Similarly, urban residents in Kenya perceived ITNs as stifling in hot climates [30].

This study revealed mixed feelings regarding the preferred shape of ITNs, influenced by gender, affordability, and comfort. It was observed that women generally preferred circular-shaped ITNs, while males preferred four-sided ones. This distinction appears to be rooted in the cultural responsibilities of females for handling and hanging the ITNs. This distinction seems to be rooted in cultural responsibilities and expectations. Similarly, a previous study reported that women are more responsible for ITN handling within households [31], which may influence their attitudes. Studies also noted that preference for ITN shape and size is differs between women and men [31,32]. According to this study, women emphasized ease of hanging and comfort, while men often prioritized cost and durability.

The study linked shape preferences to attitudes toward ITNs, warranting action by planners, although a review of literature indicated that such preferences did not significantly impact net usage. This shows the need to distinguish perceived preferences from other factors that influence actual behavior in malaria prevention for targeted intervention. [32]. In the current study, participants expressed concerns over the short sizes, likely hindering acceptance, emphasizing the need for awareness initiatives to promote ITN usage and the role of health education in its utilization [33]. Similarly, a study in Ethiopia among the school-aged population depicted that health education on malaria prevention has increased ITN utilization practice among the same population [34].

In this study, burning sensations and textile issues such as physical properties of the net fabric, including its breathability, texture, and durability, were linked to mitigated willingness to use ITN, particularly among vulnerable groups including pregnant women and children. This finding echoes previous studies that reported chemical smell and skin irritation as barriers to ITN acceptability, especially for populations with heightened sensitivity [33,35]. Despite living in a malaria-endemic area, the participants preferred not to use it during hot times. Similarly, studies revealed that high temperatures along with hot night weather are among the factors mitigating utilization [32,35–37]. These findings urge malaria control programs to address physical discomfort that is associated with ITN intervention. To ensure sustainable acceptability of ITN in the community, strategies to mitigate discomfort associated with the intervention need to be addressed.

Although ITN is mostly recommended to be used by children and pregnant women [38,39], in the current study, by the application of the ethicality construct, tension between public health intervention targets and community perception was found. While children are encouraged to sleep under ITNs, participants expressed their concerns about increasing their exposure to the chemical inappropriately. Such exposure triggers physiological responses such as vomiting and nausea, leading to heightened anxiety and stress among this population group, which further prevents the application of ITN among vulnerable populations [40].

Additionally, participating community members also raised the ethicality of compelling pregnant women to sleep under ITN. They questioned the fairness of compelling these population groups to sleep under a net that has been treated by chemicals. They expressed their fear of inhalation risks, as a pregnant woman is responsible for her health and that of her child. Similarly, another study in Ethiopia has reported reluctance of pregnant women to use ITN due to the chemical exposure and associated discomfort [38]. This underscores the need for culturally sensitive health education that can address misconceptions associated with ITN intervention. Furthermore, cultural expectations and marital norms requiring men and women to share a bed if they are married created resistance. This contributed to limited ITN distribution that requires opposite-sex siblings or family members to share a bed. This concern was raised in other studies from sub-Saharan Africa [40,41]. Thus, a malaria prevention program needs to tailor ITN intervention to community perceptions and cultural norms while also respecting household dynamics.

Like other studies [32,33,35,40], community members demonstrated a strong awareness of ITNs and their protective functions against malaria. Participants recognized that ITNs offer both physical and chemical protection; they understood that the nets are treated with chemicals that not only repel mosquitoes but also kill other disease-transmitting insects. Many noted that ITNs are more effective than alternative malaria prevention methods, such as sprays, due to their long-lasting effects, making them a cost-effective solution. However, the acceptability of ITNs is also impacted by participants’ knowledge of proper handling techniques. This highlights the necessity for clear instructions and health education from health extension workers prior to distribution, ensuring that community members are fully informed and equipped to utilize the intervention effectively [42].

Perceived effectiveness of ITN among the urban community is also one key approach in measuring acceptability to combat malaria. A study from Tanzania reads that ITN depends on its perceived effectiveness to eliminate or reduce malaria fever [43]. Malaria is among the major indirect causes of maternal and under-five children mortality [44]. Likewise, through application of the perceived effectiveness domain of TFA, the current study reveals that ITN is effective to use among children, pregnant women, new mothers, elderly individuals, and individuals with chronic diseases, contributing to several factors like weakened immunity and challenges in accessing and using malaria treatment. Similarly, studies revealed that under-five malaria is a significant factor for using ITN consistently [45,46].

Our result should be interpreted in consideration of the following limitations. First, the findings were based on qualitative data, which were collected through FGD. While this approach provides contextually rich data, it does not provide statistically generalizable results that might be inferred towards the wider population. Second, the reliance on self-reported experiences and perceptions introduces the possibility of social desirability bias, whereby participants may have emphasized culturally acceptable responses or community norms rather than their personal views [47]. This could particularly affect sensitive topics such as attitudes towards ITNs. Third, the study did not include direct observation of ITN practices, which limits the ability to validate reported behavior against actual usage patterns. Finally, the study was conducted in a malaria-endemic area during a specific malaria season; thus, we could not ensure transferability of findings across different climate contexts.

## Conclusions and recommendations

The study reveals that the community perceives insecticide-treated nets (ITNs) as ethically aligned with their values and demonstrates a solid understanding of how they function. Participants exhibited a generally positive attitude towards ITNs, despite some negative feelings regarding their height and mixed opinions about their shape. While there were reported burdens associated with ITN use, the perceived effectiveness in reducing malaria is significant. Although female participants noted increased household responsibilities due to ITNs, they also expressed confidence in integrating this intervention into their daily routines.

Overall, ITNs are accepted within the community, indicating that they are a culturally sustainable and appropriate intervention. This study explored the acceptability of ITN at the microscale level, which aids contextualized understanding and could support the design of tailored intervention to enhance ITN acceptability in the study area and other similar socio-cultural and eco-epidemiologic settings. Expanding the geographic scope of the study will help explore the diverse perspectives, experiences and contextual factors in a country where multicultural societies exist.

## Supporting information

S1 Text Interview guide. (DOCX)

S1 File COREQ checklist. (PDF)

S1 Table Framework guided content analysis. (XLSX)

## Abbreviations

ITNS: Insecticide Treated Nets
TFA: Theoretical Framework of Acceptability
FGDs: Focused Group Discussions
WHO: Word Health Organization
COREQ: Consolidated Criteria for Reporting Qualitative Research
DDT: Dichloro-Diphenyl-Trichloroethane
