# Supplementary material for: Acceptability of insecticide treated nets in an urban setting in a malarious area: Application of theoretical framework of acceptability
Source: PLOS Glob Public Health. 2026 Aug 21;6(8):e0006120. doi: 10.1371/journal.pgph.0006120 (PMC13496428; doi:10.1371/journal.pgph.0006120)
Supplement: Supplementary file 2 [file pgph.0006120.s001.docx]

**Interview Guide**

**English Version**

**Socio-demographic characteristics of the study participants**

1. Age of participants in years
2. Gender
a. Female
b. Male
3. Religion Affiliation
a. Orthodox
b. Muslim
c. Protestant
d. Catholic
e. Other
4. Marital status
a. Single
b. Married
c. Separated (No legally)
d. Divorced
e. Widow
5. Level of education
a. No formal education
b. Primary school (1-8)
c. High School (9-12)
d. Occupational Training
e. Diploma/Degree or above
6. Occupation
a. Employed
b. Unemployed
c. Retired

**Interview Guides based on theoretical framework of acceptability constructs**

1. Let us start with the definition of ITN. What does it mean? What comes to mind?
2. Why does a person supposed to use ITN?
3. What is the primary purpose of using ITN? Please let us discuss on the purpose in your specific community
4. Let us discuss on any feature of ITN to appreciate? Why?
5. Let us discuss on any difficulties while using ITN. Is there any aspect of ITN which is inconvenient? How?
6. Let us discuss on benefits and potential risks of using ITN. Is there any health concern associated with ITN? What are they?
7. Do certain groups, such as children, pregnant women, or older adult, benefit more from ITN use? How? Do you think it is fair to make them sleep under ITN every time?
8. What feeling is there about shape, texture, or smell ITN? Why is the feeling?
9. What is overall experience like when using an ITN? How does it interact with daily routine?
10. Is it demanding to hang ITN? **How?**
11. Are there specific times or situations when using the ITN feels difficult or burdensome? How
12. What would help to use the ITN more consistently or frequently? Why?
13. How does the use of ITNs align or contradicts values of the community?
14. Have you noticed any changes in the malaria situation in your area over the past few months or years? Do you believe these changes are connected to the use of ITNs? If so, how?
15. Do ITNs truly work to prevent malaria? How?

**Thank you for your time. Is there anything you would like to add? Anything we didn’t discuss?**

**Amharic Version**

የቃለ መጠይቅ መመሪያ

**የጥናቱ ተሳታፊዎች ማህበራዊ-ስነ-ሕዝብ ባህሪያት**

1. የተሳታፊዎች ዕድሜ በዓመት
2. ጾታ
a. ሴት
b. ወንድ
3. የሃይማኖት
a. ኦርቶዶክስ
b. እስልምና
c. ፕሮቴስታንት
d. ካቶሊክ
e. ሌላ
4. የጋብቻ ሁኔታ
a. ነጠላ
b. ያገባ
c. ተለያይቷል (በህጋዊ መንገድ አይደለም)
d. ተፋቷል
e. የሞተባት የሞተችበት
5. የትምህርት ደረጃ
a. መደበኛ ትምህርት የለም
b. የመጀመሪያ ደረጃ ትምህርት ቤት (1-8)
c. ሁለተኛ ደረጃ ትምህርት ቤት (9-12)
d. የሙያ ስልጠና
e. ዲፕሎማ/ዲግሪ ወይም ከዚያ በላይ
6. ሥራ
a. ተቀጥረው የሚሰሩ
b. ሥራ አጥ
c. ጡረታ የወጡ

**ዋና የጢናቱ መጠይቅ**

1. በፀረ-ተባይ የታከሙ ፀረ-ወባ መረቦች ፍቺ እንጀምር። ምን ማለት ነው? ወደ አእምሮህ የሚመጣው ምንድን ነው?
2. አንድ ሰው በፀረ-ተባይ የታከሙ ፀረ-ወባ መረቦችን ለምን መጠቀም አለበት?
3. በፀረ-ተባይ የታከሙ ፀረ-ወባ መረቦችን የመጠቀም ዋና ዓላማ ምንድን ነው? እባክዎን በማህበረሰብዎ ውስጥ ስላለው ዓላማ እንወያይበት።
4. በፀረ-ተባይ ስለ ታከሙ ፀረ-ወባ መረቦችን ማንኛውም ገጽታ እናውራ? ለምን ያን ገፅታ ወደዱት
5. በፀረ-ተባይ የታከሙ ፀረ-ወባ መረቦችን በምንጠቀምበት ጊዜ ስለሚገጥሙን ማናቸውም ችግሮች እንወያይ። የማይመች ገጽታ አለ? እንዴት?
6. በፀረ-ተባይ የታከሙ ፀረ-ወባ መረቦችን ስለመጠቀም ጥቅሞች እና ሊከሰቱ ስለሚችሉ አደጋዎች እንወያይ። ከ በፀረ-ተባይ የታከሙ ፀረ-ወባ መረቦችን ጋር የተያያዘ የጤና ስጋት አለ? እስቲ ስለእነሱ እንወያይ
7. እንደ ልጆች፣ እርጉዝ ሴቶች ወይም አዛውንቶች ያሉ የተወሰኑ ቡድኖች ፀረ-ተባይ የታከሙ ፀረ-ወባ መረቦችን በመጠቀም የበለጠ ተጠቃሚ ይሆናሉ?እንዴት? በየጊዜው በፀረ-ተባይ የታከሙ ፀረ-ወባ መረቦች ስር እንዲተኙ ማድረግ ፍትሃዊ ነው ብለው ያስባሉ? ለምን?
8. ስለ በፀረ-ተባይ የታከሙ ፀረ-ወባ መረቦች ቅርፅ፣ ሸካራነት ወይም ሽታ ምን አይነት ስሜት አለ? ለምን?
9. የፀረ-ተባይ የታከሙ ፀረ-ወባ መረቦች አጠቃቀም አጠቃላይ ተሞክሮ ምን ይመስላል? ከዕለት ተዕለት እንቅስቃሴ ጋር እንዴት ይገናኛል?
10. በፀረ-ተባይ የታከሙ ፀረ-ወባ መረቦችን መዘርጋት ከባድ ነው? እንዴት?
11. የፀረ-ተባይ የታከሙ ፀረ-ወባ መረቦች አጠቃቀም አስቸጋሪ ወይም ከባድ የሚመስልባቸው የተወሰኑ ጊዜያት ወይም ሁኔታዎች አሉ? እንዴት?
12. በፀረ-ተባይ የታከሙ ፀረ-ወባ መረቦችን በተከታታይ ወይም በተደጋጋሚ ለመጠቀም ምን ሊረዳ ይችላል?ለምን?
13. በፀረ-ተባይ የታከሙ ፀረ-ወባ መረቦችን አጠቃቀም የማህበረሰቡን እሴቶች እንዴት ያስተካክላል ወይም ይቃረናል?
14. ባለፉት ጥቂት ወራት ወይም ዓመታት በአካባቢዎ በወባ በሽታ ሁኔታ ላይ ምንም አይነት ለውጥ አስተውለዋል? እነዚህ ለውጦች በፀረ-ተባይ የታከሙ ፀረ-ወባ መረቦችን አጠቃቀም ጋር የተገናኙ ናቸው ብለው ያምናሉ? ከሆነስ እንዴት?
15. በፀረ-ተባይ የታከሙ ፀረ-ወባ መረቦች ወባን ለመከላከል በእርግጥ ይሰራሉ? እንዴት?

**ስለ ጊዜዎ እናመሰግናለን። ማከል የሚፈልጉት ነገር አለ? ያልተነጋገርንበት ነገር አለ?**

**Qajeelfama Af-gaaffii**

**Afan Oromo Version**

**Amaloota hawaas-dimoogiraafii hirmaattota qorannichaa**

1. Umurii hirmaattotaa waggaadhaan
2. Saala
a. Dhalaa
b. Dhiira
3. Amantii
a. Ortodoksii
b. Muslima
c. Pirootestaantii
d. Kaatolikii
e. Kan biraa
4. Haala gaa’elaa
   a. Qenxee
   b. Kan fuudhe
   c. Addaan bahee (Seeraan alaa) .
   d. Seeraan Hiikkaa
   e. Kan abbaan warraa/hatti warra irraa du'e
5. Sadarkaa barnootaa
a. Barnoota idilee hin qabu
b. Mana barumsaa sadarkaa tokkoffaa (1-8) .
c. Mana Barumsaa Sadarkaa Lammaffaa (9-12)
d. Leenjii Hojii
e. Dippiloomaa/Digirii fi isaa ol
6. Hojii qabachuu
a. Qaxarrii
b. Hojii dhabeeyyii
c. Soorama ba'e

**Gaaffii ijoo Qoranicha**

1. Mee hiika toora farra busaa qoricha ilbiisotaatiin wal’aanamanii irraa haa jalqabnu. Maal jechuudha? Maaltu sammuutti dhufa?
2. Namni tokko toora farra busaa qoricha ilbiisotaatiin wal’aanamanii fayyadamuu qaba? Maalif
3. Kaayyoon inni jalqabaa toora farra busaa qoricha ilbiisotaatiin wal’aanamanii fayyadamuu maali? Mee hawaasa addaa keessan keessatti haa mari'annu.
4. Mee toora farra busaa qoricha ilbiisotaatiin wal’aanamanii irratti haa mari'annu? Wa’ee isaa maal jejuu?Maaliif?
5. Yeroo toora farra busaa qoricha ilbiisotaatiin wal’aanamanii fayyadamnu rakkoolee mudatan kamiyyuu irratti haa mari'annu. Gama toora farra busaa qoricha ilbiisotaatiin wal’aanamanii kan hin mijoofne jiraa? Akkam?
6. Faayidaa fi balaa toora farra busaa qoricha ilbiisotaatiin wal’aanamanii fayyadamuu danda’u irratti haa mari’annu. ITN waliin walqabatee yaaddoon fayyaa jiraa? Maal isaan?
7. Gareen tokko tokko, kan akka daa’immanii, dubartoota ulfaa, ykn ga’eessota umuriin isaanii guddaa ta’e, itti fayyadama toora farra busaa qoricha ilbiisotaatiin wal’aanamanii irraa caalaatti fayyadamoo? Akkamitti? Yeroo hunda toora farra busaa qoricha ilbiisotaatiin wal’aanamanii jalatti akka rafan gochuun haqa qabeessa jettanii yaaddu? Maaliif?
8. Waa’ee boca, uwwisa, ykn urgaa toora farra busaa qoricha ilbiisotaatiin wal’aanamanii miira akkamiitu jira? maaliif?
9. Muuxannoon waliigalaa yeroo toora farra busaa qoricha ilbiisotaatiin wal’aanamanii fayyadamnu maal fakkaata? Hojii guyyaa guyyaa wajjin akkamitti wal qunnama?
10. Toora farra busaa qoricha ilbiisotaatiin wal’aanamanii fannisuun ulfatadha? Akkam?
11. Yeroon ykn haalli addaa toora farra busaa qoricha ilbiisotaatiin wal’aanamanii fayyadamuun rakkisaa ykn ba’aa itti dhaga’amu jiraa? Akkamitti?
12. Toora farra busaa qoricha ilbiisotaatiin wal’aanamanii haala walfakkaatuun ykn yeroo baay’ee fayyadamuuf maaltu gargaara? Maaliif?
13. Itti fayyadamni toora farra busaa qoricha ilbiisotaatiin wal’aanamanii akkamitti gatii hawaasaa walsimsiisa ykn wal faallessa?
14. Ji’oota ykn waggoota muraasa darban keessa haala dhukkuba busaa naannoo keessan jiru irratti jijjiiramni akka jiru hubattaniittuu? Jijjiiramni kun itti fayyadama toora farra busaa qoricha ilbiisotaatiin wal’aanamanii waliin walqabatee jira jettanii amantaa? Yoo jiraate akkamitti?
15. Toora farra busaa qoricha ilbiisotaatiin wal’aanamanii dhuguma dhukkuba busaa ittisuuf ni hojjetuu? Akkamitti?

**Yeroo nuuf kennitaniif galatoomaa. Wanti itti dabaluu barbaaddan jiraa? Waan nuti hin mari’anne jiraa?**
